# Missense mutations in a transmembrane domain of the *Komagataeibacter xylinus* BcsA lead to changes in cellulose synthesis

**DOI:** 10.1186/s12866-019-1577-5

**Published:** 2019-09-12

**Authors:** Luis Salgado, Silvia Blank, Reza Alipour Moghadam Esfahani, Janice L. Strap, Dario Bonetta

**Affiliations:** 1 Faculty of Science, University of Ontario Institute of Technology, Oshawa, Canada; 2 Evonik Industries AG, Rellinghauser Straße 1—11 45128, Essen, Germany

**Keywords:** K*omagataeibacter xylinus*, Pellicin, BcsA, Crystallinity

## Abstract

**Background:**

Cellulose is synthesized by an array of bacterial species. *Komagataeibacter xylinus* is the best characterized as it produces copious amounts of the polymer extracellularly. Despite many advances in the past decade, the mechanisms underlying cellulose biosynthesis are not completely understood. Elucidation of these mechanisms is essential for efficient cellulose production in industrial applications.

**Results:**

In an effort to gain a better understanding of cellulose biosynthesis and its regulation, cellulose crystallization was investigated in *K. xylinus* mutants resistant to an inhibitor of cellulose I formation, pellicin. Through the use of forward genetics and site-directed mutagenesis, A449T and A449V mutations in the *K. xylinus* BcsA protein were found to be important for conferring high levels of pellicin resistance. Phenotypic analysis of the *bcsA*^*A449T*^ and *bcsA*^*A449V*^ cultures revealed that the mutations affect cellulose synthesis rates and that cellulose crystallinity is affected in wet pellicles but not dry ones.

**Conclusions:**

A449 is located in a predicted transmembrane domain of the BcsA protein suggesting that the structure of the transmembrane domain influences cellulose crystallization either by affecting the translocation of the nascent glucan chain or by allosterically altering protein-protein interactions.

**Electronic supplementary material:**

The online version of this article (10.1186/s12866-019-1577-5) contains supplementary material, which is available to authorized users.

## Background

Cellulose, an abundant glucan polymer, is synthesized by organisms belonging to a variety of phylogenetic domains [1]. In nature, the majority of plants and the bacterium *Komagataeibacter xylinus* (formerly *Gluconacetobacter xylinus*), synthesize a crystalline form designated cellulose I [2]. The glucan chains in cellulose I are arranged parallel to one another and assembled side-by-side forming structures known as microfibrils [3, 4]. Cellulose I exists in two allomorphs; Iα (triclinic) and cellulose Iβ (monoclinic), which can be differentiated using solid-state ^13^C NMR [5]. The two allomorphs are distinguished from one another by their crystal packing hydrogen bond interactions and molecular conformation, which alter the physical characteristics of the polymer [6]. One cellulose microfibril may contain both types of allomorphs; with the Iβ form dominant in higher plants and the Iα form dominant in bacteria and algal species [7]. An alternative form of cellulose, known as cellulose II, can be obtained through either industrial mercerization (alkali treatment) or regeneration (solubilization and subsequent recrystallization) [5]. In cellulose II, the glucan chains are arranged in an antiparallel conformation [8]; each glucose residue has an additional hydrogen bond which makes it a more thermodynamically stable conformation. In contrast to cellulose I, cellulose II is made by very few organisms, such as the gametophyte cells of the marine alga of the genus *Halicystis* and gram-positive bacteria of the genus *Sarcina* [1].

In bacteria, cellulose is synthesized as an extracellular polysaccharide via a single polymerization step that uses uridine diphosphoglucose (UDP-glucose) as substrate which undergoes self-assembly at the site of biosynthesis [9]. This process is driven by integral membrane glycosyltransferases (GTs) that couple the elongation of the polymer with the translocation of the product outside the cell [10]. A single cell of *K. xylinus* can polymerize up to 200,000 glucose molecules into β-1,4 glucan chains every second [11]. The synthesis of bacterial cellulose (BC) is precisely regulated by a diverse number of enzymes and regulatory proteins. Synthesis occurs in the periplasm by cellulose synthesizing complexes (CSCs) or terminal complexes (TCs), which are associated with outer membrane pores organized linearly along the long axis of the cell [12–14]. Protofibrils are thought to be assembled into ribbon-shaped microfibrils that elongate while remaining associated with the cell envelope, even during cell division, giving rise to BC or pellicle [14]. The polymerization of cellulose is catalyzed by cellulose synthase (CS) without the formation of intermediates [15].

*K. xylinus* possesses a cellulose synthase operon (*bcs* operon) composed of three to four genes, depending on the strain or subspecies: *bcsAB* (or *bcsA* and *bcsB*), *bcsC*, and *bcsD* [16]. The protein product encoded by *bcsA* is an integral membrane protein that contains multiple transmembrane (TM) domains; four N-terminal and four C-terminal TM helices separated by an extended intracellular loop between helices 4 and 5 that forms the glycosyltransferase (GT) domain [10] that includes a conserved motif with three spaced aspartates followed by a pentapeptide sequence motif (QxxRW) characteristic among processive GTs like hyaluronan, chitin and alginate synthases [16, 17]. The GT domain of BcsA contains seven β-strands surrounded by seven α-helices. The narrow channel for cellulose translocation is formed by the TM helices 3 to 8 and appears to accommodate 10 glucose residues of the translocating glucan chain [10]. Most inverting GTs require an essential divalent cation for catalysis. The cation is coordinated by the conserved Asp-X-Asp (DXD) motif at the active site to stabilize the nucleotide diphosphate leaving group during glycosyl transfer [10]. The activity of BcsA is stimulated by an allosteric regulator, 3′, 5′-cyclic diguanylic acid (c-di-GMP), which interacts with the PilZ domain located at the C-terminus of the BcsA, next to the GT domain [18, 19]. Binding of the c-di-GMP causes a conformational change in the PilZ domain disrupting a specific Arg-Glu salt bridge, which results in the displacement of a “gating loop” that allows UDP-glucose to bind [19].

In contrast, the product of the *bcsB* gene is a predominantly β-stranded periplasmic protein with a single transmembrane anchor that interacts with BcsA [10]. The BcsB protein contains four domains: two jellyroll domains, which show high similarity to carbohydrate binding domains (CBDs), and two flavodoxin-like folds [10]. Although BcsB is essential for catalysis, only the interactions of its C-terminal TM anchor and an amphipathic helix seem to be necessary to stabilize the TM region of BcsA for catalysis [19]. In addition, BcsB is thought to be required to guide cellulose across the periplasm toward the outer membrane via the two carbohydrate-binding domains (CBDs) [10]. While the functions of BcsC and BcsD have not been fully elucidated, they both influence cellulose crystallinity. For example, BcsC is predicted to form a β-barrel porin located in the outer membrane, which is preceded by a relatively extensive periplasmic domain carrying tetratricopeptide repeats which may be involved in complex assembly [20, 21]. On the other hand, BcsD, for which the crystal structure has been elucidated, is thought to function as a periplasmic channel for the nascent cellulose strand [22, 23]. Indeed, the isolation of *K. xylinus bcsD* mutants, which form smooth colonies and produce reduced amounts of cellulose in culture, suggests that BcsD is involved in cellulose crystallization [9]. Recently, Sajadi et al. [24] showed that heterologous expression of *bcsD* in *Escherichia coli* increased the crystallinity but did not affect the yield of BC. In addition to the cellulose synthesis operon genes, ancillary genes have been implicated in cellulose biosysnthesis. One of these genes, *bcsZ* (formerly *cmcax)*, which is located upstream of the cellulose synthase operon, encodes an endo-β-1,4-glucanase (BcsZ, formerly CMCax) which possesses cellulose hydrolyzing activity that is essential for BC production [25]. In addition, *bcsH* (formerly *ccpax)*, also located upstream of the *bcs* operon, encodes a cellulose complementing protein. The function of BcsH is not well understood, but it has been suggested to be important for cellulose crystallization [26]. Mutations in the *bcsH* locus result in a significant decrease in cellulose production [27]. A downstream region of the operon which encodes an enzyme with exo-1,4-β-glucosidase activity (BglAx) towards cellotriose or larger cello-oligosaccharides also leads to significant decrease in cellulose production when mutated [28]. The precise role of BglAx is, however, still unknown [27].

In a previous study, we used a chemical genetic approach to identify pellicin, a potent inhibitor of cellulose pellicle formation that exerts its effect extracellularly [29]. An important property of pellicin is that it can inhibit cellulose crystallization without disrupting cell growth [29]. In addition, pellicin does not inhibit cellulose synthase activity directly since crude membrane preparations from *K. xylinus* cells grown in pellicin-containing medium show increased cellulose synthesis compared to untreated membrane preparations [29]. These and other features of pellicin suggest that it allows normal cellulose II biosynthesis assembly but inhibits the assembly of cellulose I. In order to better understand the mechanism of action of pellicin, we have conducted a forward genetic screen aimed at identifying pellicin-resistant mutants. Our current findings indicate that in *K. xylinus,* one indirect target for pellicin activity is the BcsA protein*.*

## Results

### Forward genetic screening identifies mutant with resistance to pellicin

Crystallinity of cellulose pellicles formed by *K. xylinus* is greatly reduced in the

presence of pellicin [29]. In an effort to further investigate cellulose crystallization during biosynthesis, we conducted a forward genetic screen aimed at identifying *pellicin resistant* (*plr*) mutations. To do this, we mutagenized a *K. xylinus* ATCC53582 culture with ethyl methanesulfonate (EMS); 4 million mutagenized cells were initially screened in medium containing 10 μM pellicin. Among the mutants that were isolated, which exhibited pellicin resistance to 5 μM (*plr1-plr10, plr1*2) or 10 μM pellicin (*plr11*), three (*plr13, plr14, plr15*) were able to produce a thick pellicle at concentrations of pellicin 150–170 μM pellicin. Among these *plr* mutants, we focused on *plr15* for further analysis, as it had the highest resistance (170 μM).

As a measure of crystalline cellulose, we determined the cellulose content of *plr15* and wild-type pellicles formed in the absence and presence of 10 μM pellicin from static cultures. Total cellulose content was measured using an acetic/nitric acid extraction which solubilizes all sugar polymers with the exception of crystalline cellulose [30]. The pellicles produced by *plr15* under these conditions were significantly reduced (approximately 23%) compared to those formed in SH medium lacking pellicin, indicating that *plr15* is not pellicin-blind (Fig. 2). Although this analysis does not provide structural information about the cellulose formed by *plr15*, it does suggest that the mutation has an effect on crystalline cellulose production in the presence of pellicin.

The growth rate of *plr15* was not significantly affected in medium containing pellicin, indeed the growth rate was identical to that of wild type cultures grown in medium lacking pellicin (Additional file 1). However, the final optical density of wild type grown cells in medium containing pellicin was significantly higher (OD600 = 1.242 ± 0.06) compared to the final optical density in medium lacking pellicin (OD600 = 1.006 ± 0.06). Based on this observation, the mutation in *plr15* does not affect growth rate, but the higher final density observed only in the pellicin-treated wild type cultures suggests a connection between density and crystalline cellulose production.

### The *plr15* mutation maps to the catalytic cellulose synthase gene, *bcsA*

In order to identify the causative mutation conferring pellicin-resistance in *plr15*, we sequenced the *bcs* operons from wild type and *plr15* genomic DNA. DNA sequence comparisons of the *bcs* operon genes, revealed a single point mutation in the catalytic cellulose synthase gene, *bcsA,* of *plr15,* at position 1345 where a guanine is substituted to adenine. This base mutation is predicted to cause a change from a non-polar alanine residue (A) to a polar threonine residue (T) at position 449 of the amino acid sequence (A449T). No other mutations were found in the remaining *plr15 bcs* operon gene sequences*.*

To confirm that the A449T missense mutation in BcsA was indeed the causative mutation for the observed pellicin resistance, the mutation was recreated in wild-type DNA from *K. xylinus* ATCC53582 by site-directed mutagenesis. Allele replacement of this mutated version of *bcsA* (*bcsa*^*A449T*^) was achieved through homologous recombination in wild type cells using a modified version of the vector pKNG101 [31]. The pKNG101 plasmid has the advantage of having a streptomycin-resistance gene as a selectable marker for the cointegration event as well as the *Bacillus subtilis sacB* gene as a counter-selectable marker to improve a second crossover event [31]. Following selection for streptomycin resistance and counterselection on sucrose, eight colonies were able to produce a pellicle in the presence of pellicin. The sequence of the *bcsA* DNA from three of these colonies revealed a substitution of nucleotide G to A at position 1345 of the gene. This base mutation replicated the one identified in *plr15*. DNA sequence analysis of the other five pellicle-producing colonies revealed a substitution of nucleotide C to T at position 1346 of the nucleotide sequence of the *bcsA* gene. This base substitution is predicted to change the alanine residue (A) to a valine residue (V). It is likely that this mutation was the result of a polymerase error during the PCR. Although not originally planned, the generation of this mutant provides additional confirmation that this specific alanine is important for pellicin resistance. Sequencing of *bcsA* DNA from 10 colonies that did not produce a pellicle in the presence of pellicin revealed no nucleotide changes. The pellicin-resistant phenotype observed in both *bcsA*^*A449T*^ and *bcsA*^*A449V*^ mutants suggest that the A449 residue is responsible for the observed difference in pellicin sensitivity.

We confirmed that the newly generated mutants phenocopied the original mutant by growing them in broth cultures in the presence of 10 μM pellicin. The newly created *bcsA*^*A449T*^ and *bcsA*^*A449V*^ mutants displayed identical pellicle-forming capacity to the original *plr15* mutant (Additional file 2 a). Interestingly, when grown in medium containing high concentrations of pellicin (50 or 100 μM), *plr15* and *bcsA*^*A449T*^ formed pellicle more slowly than *bcsA*^*A449V*^ (Additional file 2 b and c). However, *plr15* as well as the *bcsA*^*A449T*^ and *bcsA*^*A449V*^ mutants eventually (after 10 days) formed thick pellicles comparable to those formed at a lower pellicin concentration.

**Fig. 1:**
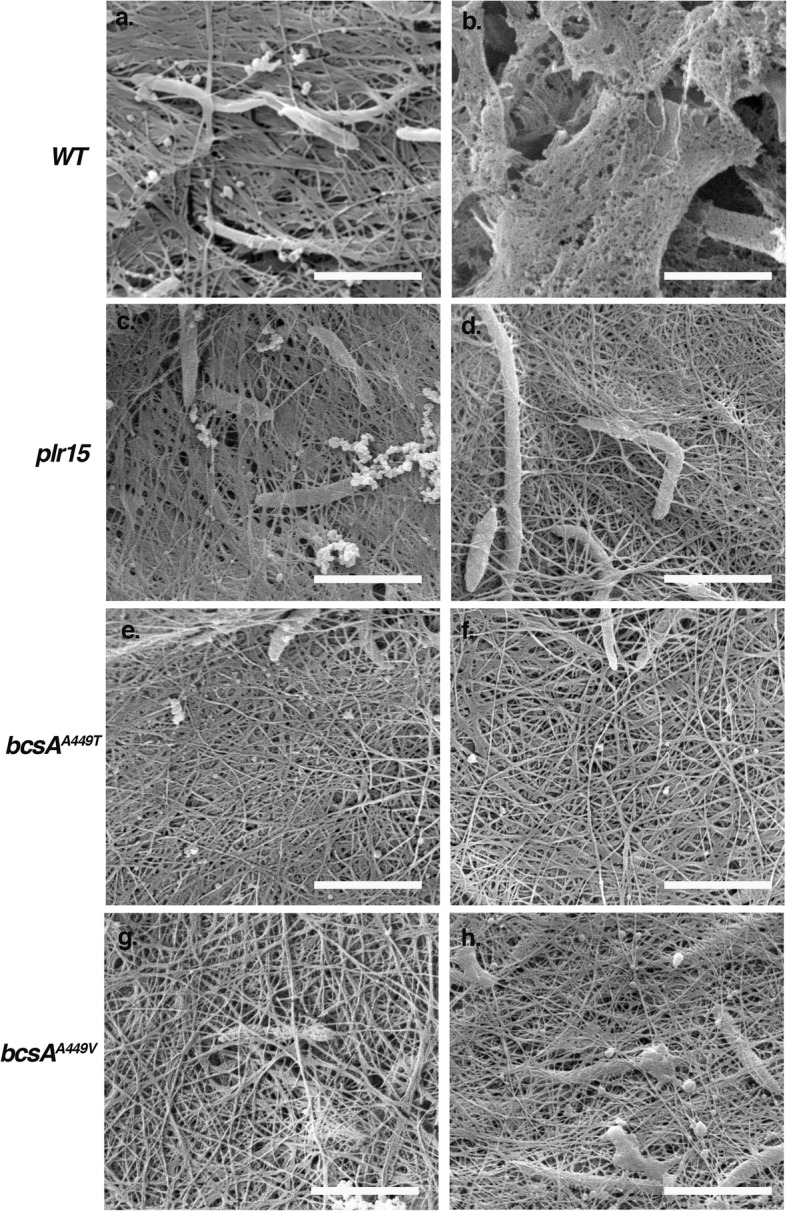
Scanning electron micrographs of pellicles from *K.xylinus* wild-type (WT), *plr15*, *bcsaA449T*, or *bcsaA449V* cultures grown for seven days under static conditions in the absence (**a**, **c**, **e**, and **g**) or presence of 10 μM pellicin (**b**, **d**, f, and **h**). In contrast to wild type cells which produce no pellicle in the presence of pellicin and are embedded in a gel-like substance (**b**), pellicles produced by mutant cells are surrounded by long cellulose fibrils (**c**, **d**, **e**, **f**, **g**, and **h**). Bars correspond to 2.5 μm

**Fig. 2:**
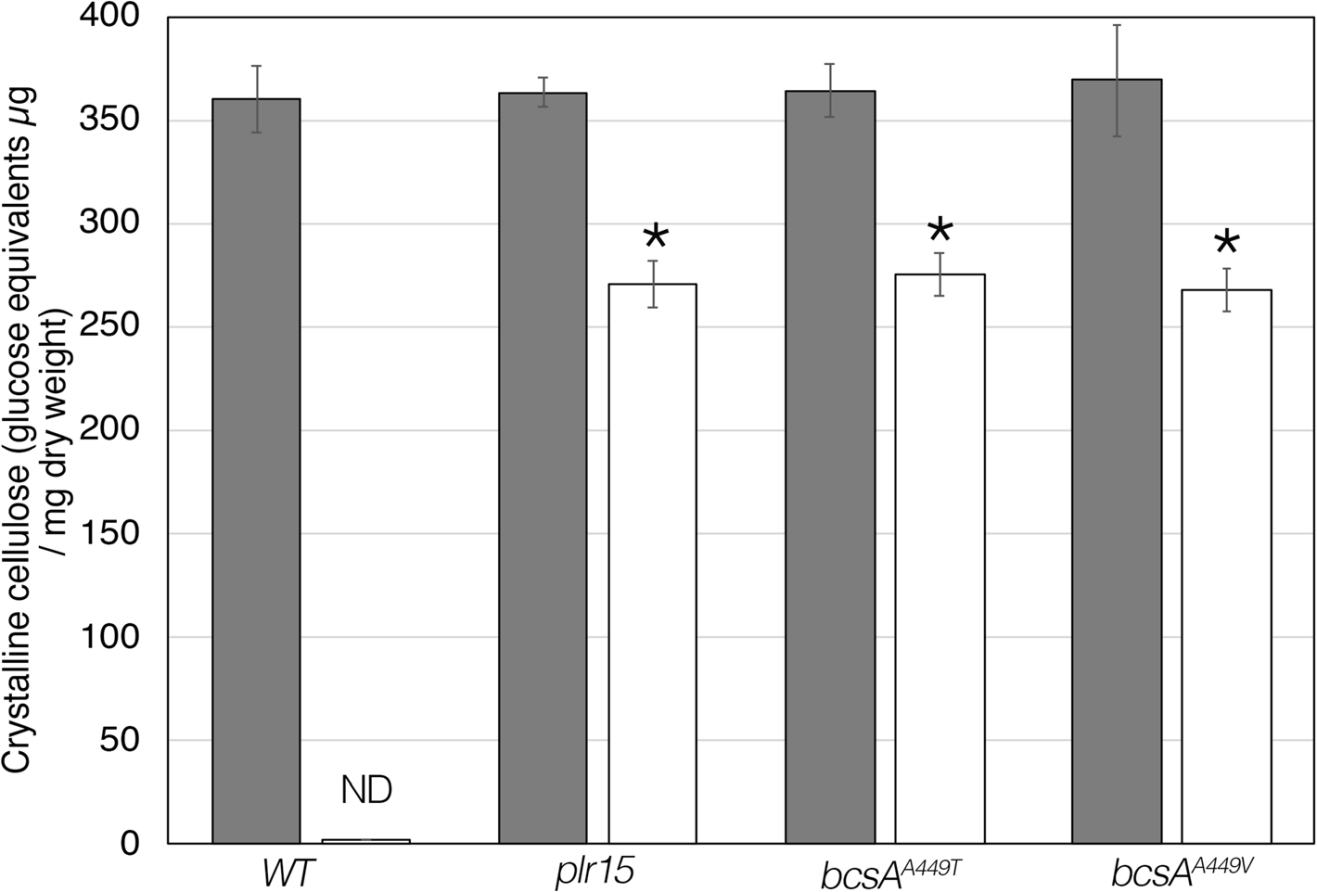
Crystalline cellulose formed by *K. xylinus* wild type, *plr15*, *bcsA*^*A449T*^, and *bcsA*^*A449V*^. The crystalline cellulose content of pellicles from wild type (WT), *plr15*, *bcsA*^*A449T*^, and *bcsA*^*A449V*^ mutants in SH (gray bars) or SH + pellicin (white bars) determined by the method described by Updegraff [30]. The crystalline cellulose is expressed as glucose equivalents per dry weight (μg/mg). Values are the mean ± standard deviation of three biological replicates. ND denotes that no glucose equivalents were detected. Asterisks indicate statistically significant differences between pellicin-treated and pellicin-untreated cultures according to one-way ANOVA (*p* < 0.05)

The appearance of the pellicles under high magnification using scanning electron microscopy (SEM) indicate that in contrast to wild type cells (Fig. 1a and b), mutant pellicles produced in the presence of pellicin (Fig. 1d, f, h) contain long cellulose strands that are indistinguishable from those produced in the absence of pellicin (Fig. 1c, e, g). Similarly, *bcsA*^*A449T*^and *bcsA*^*A449V*^ produced less but significant amounts of crystalline cellulose (approximately 25% less) in the presence of pellicin, indicating that the mutations had comparable effects on cellulose production to *plr15* (Fig. 2).

**Fig. 3:**
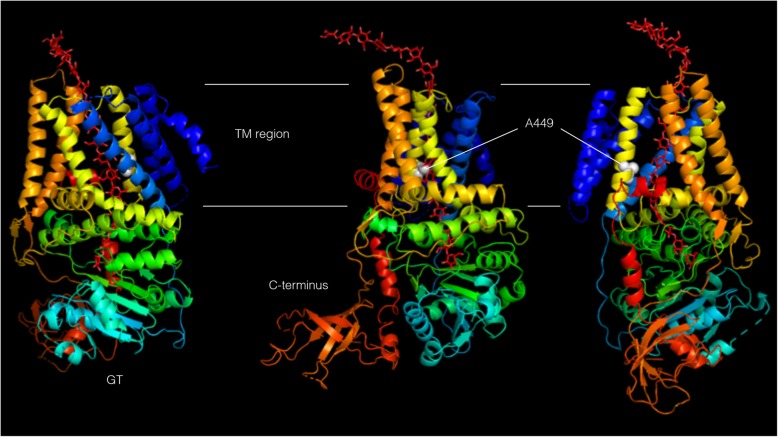
Architecture of the *K. xylinus* BcsA protein modeled after the *Rhodobacter sphaeroides* cellulose synthase template PDB 4hg6A. The location of alanine at position 449 is indicated by the white spheres and nascent glucan chain is shown in red. The model is coloured based on rainbow colouring scheme with the N-terminus coloured blue and C-terminus coloured red. Each image is rotated 90° relative to the preceding image

To better understand how the missense mutations might affect BcsA function, we mapped the position of the mutations onto a three dimensional model of KxBcsA based on the crystal structure of *Rhodobacter sphaeroides* BcsA [10]. According to the 3D model, the substituted 449 alanine is predicted to be in the middle of the sixth transmembrane helix of the protein (Fig. 3). At this position, the alanine residue is predicted to be in close proximity to the translocating glucan as part of the narrow channel accommodating the nascent glucan chain.

### *plr* mutations affect cellulose synthesis rates

**Fig. 4:**
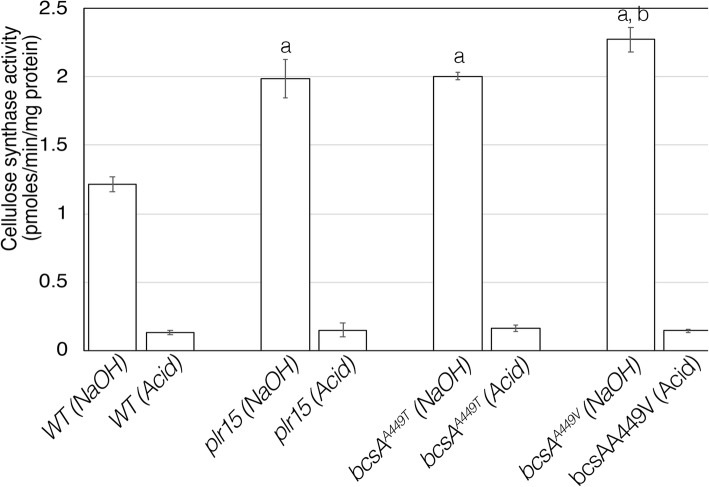
In vitro cellulose synthase assay using membrane preparations of *K. xylinus* wildtype and *plr* mutant cells with UDP-[^3^H]glucose as substrate. Mutants show an increased cellulose synthase activity. Values are the mean ± standard of three experimental determinations. Letters indicate statistically significant differences compared to wild type (**a**) or compared to *plr15* and *bcsA*^*A449T*^ (**b**) according to two-tailed student t-test (p < 0.05)

**Fig. 5:**
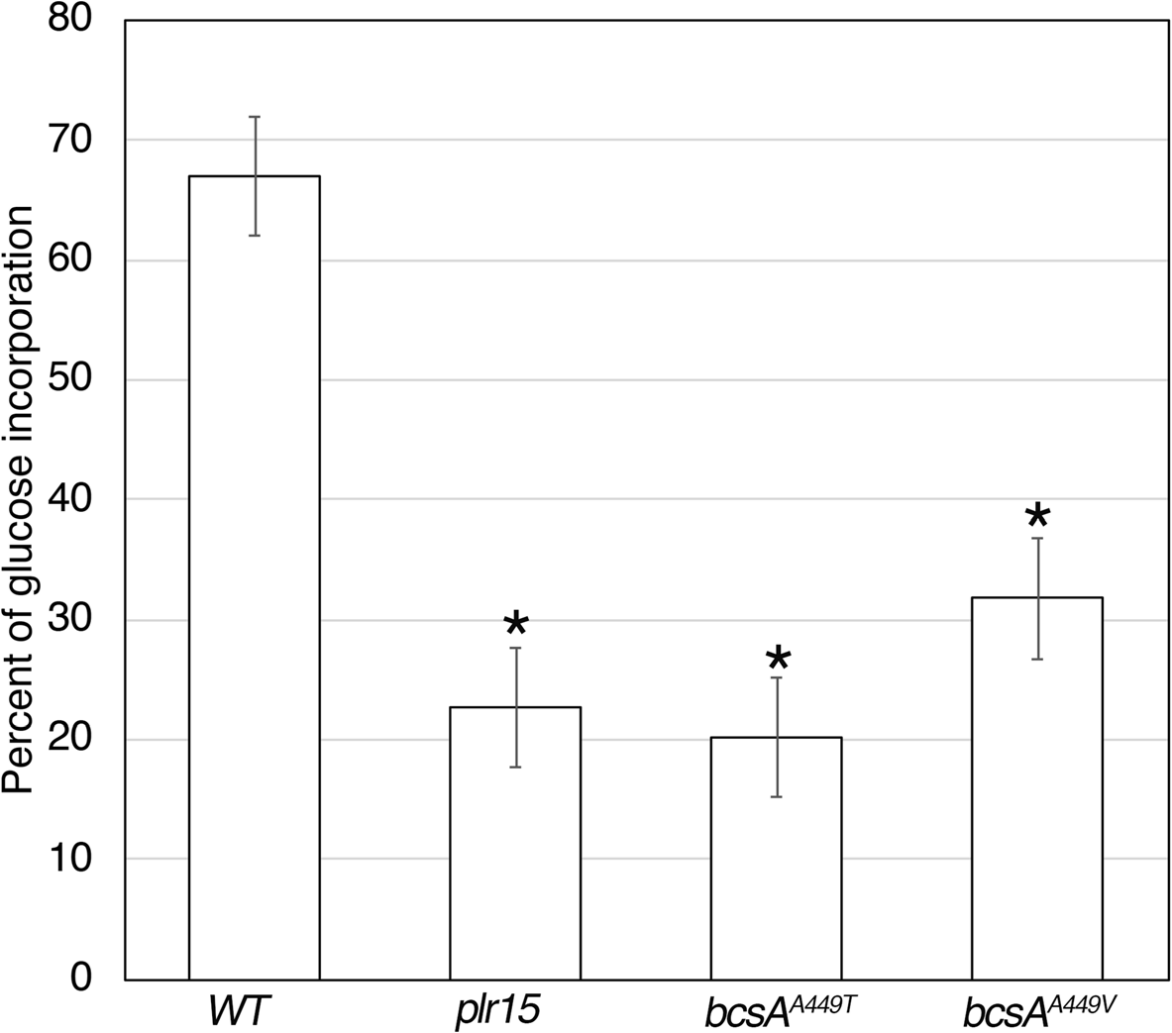
Comparison of radiolabeled glucose incorporation into the cellulose fraction of pellicles from *K. xylinus* wild type and *plr* mutants. Measurements were determined after bacterial cultures were incubated in ^14^C-glucose for five hours. The vertical axis is expressed as percentage of ^14^C-glucose detected in the insoluble fractions divided by the soluble plus insoluble fractions. Asterisks indicate statistically significant differences compared to wild type according to two-tailed student t-test (p < 0.05). Values are the mean ± standard deviation (*n* = 6)

**Fig. 6:**
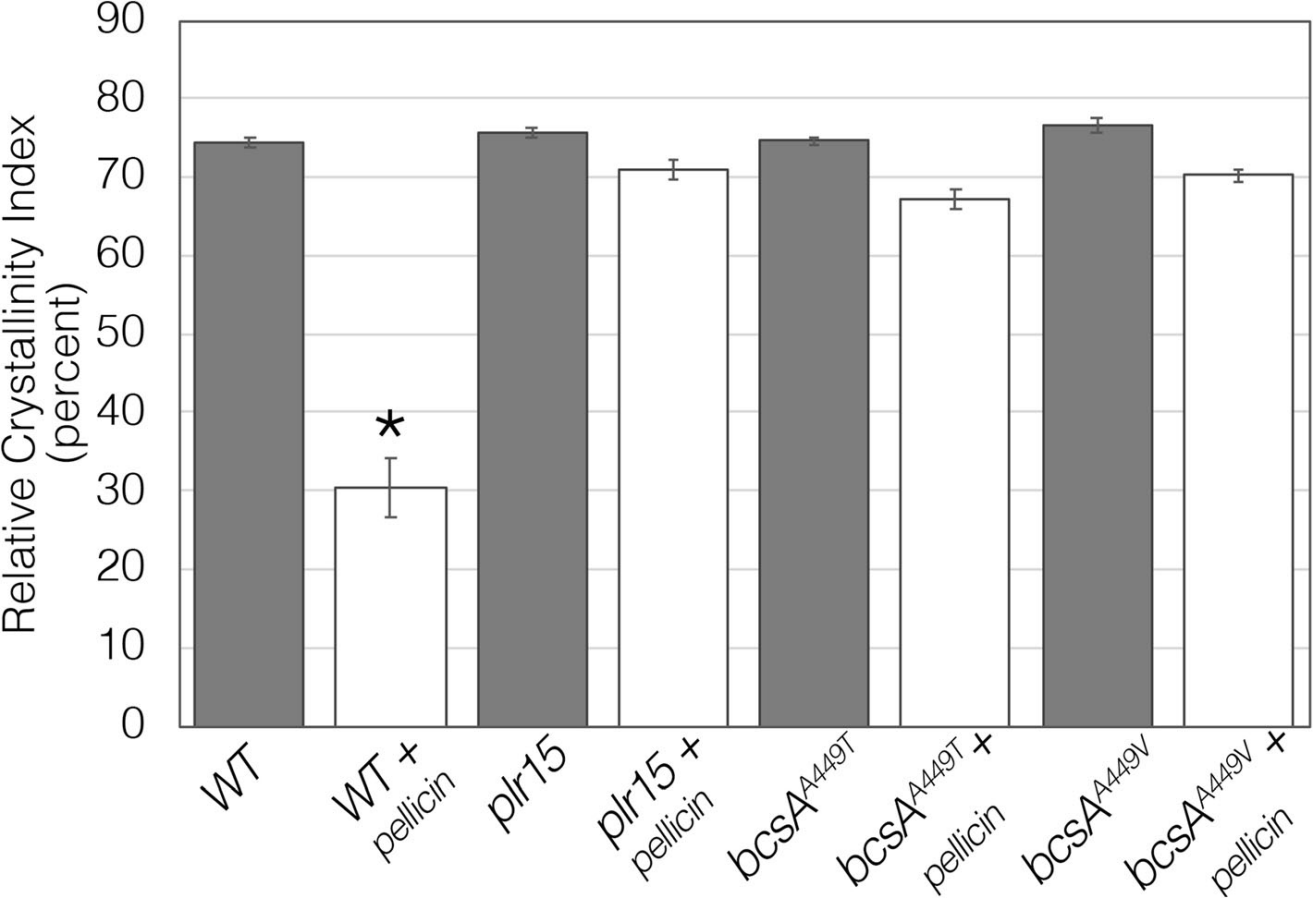
Relative crystallinity index (RCI) of extracellular cellulose produced by *K. xylinus* cultures grown in the absence (grey) or presence of 10 μM pellicin (white) based on powder X-ray diffraction. Asterisk indicates statistically significant difference compared to wild type according to two-tailed student t-test (*p* < 0.05). Values are the mean ± SD (*n* = 3)

xTo determine whether *plr* mutations had any consequences on cellulose synthase activity, crude membrane preparations from wild-type and mutant cultures were used to assay the in vitro rates of ^3^[H]-UDP-glucose incorporation into cellulose. The mutants exhibited a significant increase (almost double) in the cellulose synthesis rates compared to wild type (Fig. 4). The incorporation of UDP-glucose measured in a standard assay represents both crystalline and none crystalline cellulose. Reactions treated with 0.5 M NaOH (NaOH) contain both cellulose I and cellulose II. Acetic-nitric acid (Acid) treatment of reactions removes cellulose II while retaining cellulose I. When the assay products were treated with an acetic/nitric acid mixture instead of 0.5 M NaOH, the incorporation rates were low and comparable to those of wild-type, indicating that most of the product measured using standard assay conditions is non-crystalline cellulose (Fig. 4). In order to determine if *plr* mutants produce more or less crystalline cellulose, we measured ^14^[C]-glucose incorporation into the cellulose of whole cells grown under static conditions over a longer time frame (5 h). In contrast to the first assay, *plr* mutant cultures produce significantly less crystalline cellulose than wild-type cultures (Fig. 5). The mutants exhibit reduced radiolabeled glucose incorporation into the acid-insoluble fraction compared to the wild type.Taken together, these results suggest that the higher rates of cellulose synthesis are the result of a reduction in cellulose crystallinity rather than an altered catalytic activity of the enzyme, BcsA. Since these effects are in contrast to the crystalline cellulose measurements obtained using dried pellicles as described above, we also measured cellulose crystallinity by powder X-ray diffraction (XRD) to determine if the reduced cellulose crystallinity was a feature of hydrated cellulose. Cellulose crystallinity indices of *plr* pellicles obtained by XRD were comparable to untreated, wild type pellicles (Fig. 6), indicating that dried pellicles contain crystalline cellulose I. In contrast, when pellicles are produced by cultures grown in the presence of pellicin, wild type cultures produce non-crystalline cellulose II instead of cellulose I, while *plr* mutants produce crystalline cellulose I under these conditions.

## Discussion

Cellulose biosynthesis in *K. xylinus* is a complex, poorly understood process involving an assemblage of proteins [32, 33]. In an effort to create additional tools for elucidating this process, we had conducted a chemical genetics screen to identify molecules that impinge on the production of crystalline cellulose in *K. xylinus,* which resulted in the identification of the cellulose biosynthesis inhibitor, pellicin [29]. In the present study, we have gained insights into the mode of action of this molecule by isolating mutations that confer resistance to its inhibitory effect on cellulose pellicle formation. The pellicin-resistant mutations lead to the production of comparable amounts of crystalline cellulose when cultures were grown in the absence of pellicin and about 80% of the wild type levels of crystalline cellulose when pellicin was present in the growth medium.

Cloning the causative mutation of the pellicin-resistant mutant, *plr15,* revealed that an A449T substitution in the BscA protein was involved in pellicin resistance. This was confirmed by site-directed mutagenesis which recreated the same mutation in wild type *K. xylinus* cells. During the course of generating the A449T mutation, we also identified mutants with an A449V substitution, which resulted in mutants that are phenotypically similar to those with the threonine substitution. In this regard, the A449T mutation resulted in a non-polar to polar substitution and the A449V mutation in a non-polar to a non-polar substitution; therefore, it seems that the polarity of the substitution at the A449 position of BcsA is not the determining factor for conferring pellicin resistance, rather it is more likely a result of the bulkier side chain of valine compared to the wild type alanine.

In order to localize the alanine 449 residue in BcsA, we mapped the A449 position onto a 3D model of the BscA protein. Alignment with the BcsA protein from *Rhodobacter sphaeroides* revealed that the alanine 449 residue maps to the sixth transmembrane domain (TMH6) of the protein. TMH6 of BcsA forms part of the transmembrane pore which allows the translocation of the nascent glucan chain from the plasma membrane into the periplasm [10]. It has been shown that molecules that disrupt cellulose crystallization, such as calcofluor and pellicin, also cause an increase in the rate of glucose polymerization indicating that polymerization and crystallization are coupled processes [29, 34, 35]. Interestingly, calcofluor-induced non-crystalline cellulose is only observed in the wet state and reverts to crystalline cellulose I in the dried state [35]. Interpretation of this result is that calcofluor disrupts microfibril assembly in the wet state which is reversed as the hydrogen bonds between the glucan chains form upon drying [35]. These observations are supported by the crystal structures of BcsA-BcsB from *R. sphaeroides* which predict that glucan chain extension and translocation are coupled [10, 33]. This implies that the rate of polymerization is limited by the time necessary for emerging glucan chains to hydrogen bond as well as by glucan translocation. In agreement with these observations, the consequences of missense *plr* mutations at A449 confer not only resistance to pellicin, but also cause an increase in the rate of glucose polymerization further implying that the cellulose is less crystalline. Furthermore, at least in the short term, the mutations also cause a decrease in the amount of glucose that is incorporated into crystalline cellulose. In dried *plr-*derived pellicles, however, the total amount of crystalline cellulose is comparable to that of wild-type.

In *K. xylinus,* altering the interactions between amino acids of TMH6 in *plr* mutants and the translocating glucan may change the configuration of the nascent chain prior to crystalline microfibril assembly. This may in turn, cause collateral effects in crystalline cellulose production. Another way in which altering the translocation process may disrupt cellulose crystallization involves the geometry of the glucan chain as it is extruded from the BscA-BscB complex. In particular, the glucan exits the complex between loops 5/6 and 7/8 at the BcsA-BcsB interface, towards the periplasm, twisting so that it protrudes laterally from the complex [10]. The precise extrusion microenvironment could be indispensable for the proper interaction between the nascent glucan and other proteins in the periplasm required for cellulose crystallization. Therefore, if pellicin were able to alter the ability of an accessory protein to interact with the TM region of BcsA, it could also affect glucan extrusion thereby hindering the overall crystallization process.

By analogy to the plant *Arabidopsis*, Harris et al. [36] have shown that resistance to the cellulose biosynthesis inhibitor, quinoxyphen, is the result of mutations in the *bcsA*-homologous gene, *CESA1.* Interestingly, a missense mutation in CESA1 causing an A903V substitution leads to quinoxyphen resistance as well as reduced crystalline cellulose content and crystallite size [36]. In addition, the velocity of cellulose synthase complex movement across the plasma membrane is increased in the mutant, suggesting an increase in the rate of polymerization [36]. The *cesa1*^*A903V*^ mutation aligns with the tyrosine 455 residue of BcsA in *R. sphaeroides* which likely interacts with the translocating glucan [10]. In *K. xylinus*, the latter residue aligns in the TMH6 with a histidine 445 residue, which is positionally upstream of the alanine 449 residue. The A449 substitutions have similar effects to the A-V substitution observed in the *Arabidopsis* orthologue, therefore, it is plausible that pellicin disrupts cellulose synthesis in a similar manner to quinoxyphen, by indirectly disrupting glucan chain translocation. If this was the case, it implies that the process of glucan translocation in both domains is a general feature of cellulose synthesis and can have a profound effect on crystalline cellulose production.

## Conclusion

In this study we have shown that the cellulose synthase gene (bcsA) not only plays a role in the catalytic polymerization process, but also in the process of crystalline cellulose I production. Missense mutations A449T and A449V in BcsA, which confer high levels of resistance to the cellulose I inhibitor, pellicin, are located in the sixth transmembrane spanning domain of BcsA and are part of the glucan translocating channel. Based on our results, we infer that pellicin inhibits cellulose I formation by impeding proper cellulose translocation of the glucan within the polysaccharide channel of the BcsA protein. It is possible that pellicin exerts its effects by impinging a CS accessory protein involved in cellulose crystallization and that changes to BcsA as a result of the A449T and A449V mutations counteract this effect by conferring greater functionality to the BcsA protein or by stabilizing protein-protein interactions.

## Materials and methods

### Bacteria, medium and growth conditions

All strains of *Komagataeibacter xylinus (*ATCC 53582) were cultured in liquid or on solid Schramm-Hestrin (SH)/agar medium at 30 °C. For preparations requiring cells free of cellulose, 0.3% (v/v) cellulase from *Trichoderma reesei* ATCC 26921 (Sigma-Aldrich) was added to the culture medium 24 h before harvesting to digest the cellulose and obtain a uniform suspension. *E. coli* strains used in this study (DH5α, 5-alpha F′ *Iq*, and Mach1 *pir116*+), were grown in Luria-Bertani (LB) broth at 37 °C under static conditions or on a rotatory shaker (250 rpm). For selection of resistant markers, antibiotics were used at the following concentrations: kanamycin, 50 μg/mL; ampicillin, 100 μg/mL; and streptomycin, 50 μg/mL. The *E. coli* strain Mach1 *pir116+* was kindly provided by Dr. Ayush Kumar (University of Manitoba), whereas DH5α, 5-α F′ *Iq*, and *dam−/dcm −* strains were from New England Biolabs.

### EMS mutagenesis and mutant isolation

Wild type cells were mutagenized by treating 3.2 × 10^7^ cells/ml with 140 mM ethyl methane sulfonate (EMS) for 50 min at 30 °C. This resulted in survival of ~ 1.6 × 10^7^ cells/ml. Approximately 4 million mutagenized cells were used to seed four 96-well plates with 0.2 mL SH containing 10 μM pellicin. The wells were monitored for the presence of a pellicle after 5 days incubation at 30 °C under static conditions. Pellicles that formed were collected and digested with cellulase, diluted and used to inoculate another four sets of 96-well plates. This was repeated one more time and pellicle forming cells were diluted and spread-plated on solid medium containing 10 μM pellicin to isolate individual colonies that were resistant to pellicin. In total, 18 isolates that showed consistent pellicin resistance were retained for further analysis.

### Preparation of cultures to determine the degree of pellicin resistance

Cells from a single colony were first cultivated in SH liquid medium on a rotary shaker at 30 °C for 7 days. A cellulose-free uniform suspension cells was obtained by adding cellulase to the growth medium at a final concentration of 0.3% (v/v). The initial inoculum was prepared as described above. The presence or absence of a pellicle was assessed after 7 days under static conditions at 30 °C. Each well of a 96-well plate was loaded with a total volume of 200 μL of bacterial culture containing either dimethylsulfoxide (DMSO) or pellicin dissolved in DMSO; pellicin concentrations ranged from 5 to 200 μM. The results are representative of nine technical replicates obtained from three biological replicates.

### Growth kinetics of wild-type and *plr* cultures

In order to determine the effect of pellicin on the growth of *K. xylinus* wild.

type and the *plr15* mutant, cultures were examined in SH medium containing or lacking 10 μM pellicin. Growth kinetic data was collected from cultures grown in 96-well microtitre plates inoculated with a total volume of 200 μL SH medium containing 0.3% (v/v) cellulase and either DMSO or pellicin dissolved in DMSO. The inoculum was prepared by harvesting 5-day old, cellulase-digested cultures by centrifugation at 17,000 x *g* for 5 min at room temperature, followed by three washes with fresh SH medium. The starting inoculum was adjusted to an optical density at 600 nm (OD600) of 0.02 in SH broth. The bacterial culture plates were incubated at 30 °C with shaking at 150 rpm. The optical density was measured using Bio-Rad xMark™ Microplate Absorbance Spectrophotometer (Bio-Rad Laboratories Ltd., Mississauga, ON). Bacterial growth was monitored for 154 h. The data from eight technical replicates obtained from two biological replicates were averaged and used for statistical analysis.

### Determination of crystalline cellulose content

The crystalline cellulose from pellicles formed by *plr* mutants and wild type was determined as previously described [30]. Briefly, inocula from *plr15* and wild type were prepared as described above. Pellicles from 7 day-old cultures were harvested, treated with 0.1 N NaOH at 80 °C for 20 min to lyse cells, neutralized by shaking in ultra-pure water for 24 h with two water changes, washed with acetone five times and air-dried at room temperature for 10 days. The dried pellicles were weighed and 15 mg of each pellicle transferred to a glass test tube. Each pellicle fraction was soaked in 3 mL of Updegraff solution (1:2:8 acetic acid:water:nitric acid) in a boiling water bath for 1 h. The acid resistant pellicles were transferred to pre-weighed Whatman GF/A glass fibre filters and washed several times with 70% ethanol by vacuum. These insoluble cellulose fractions were then allowed to dry overnight at room temperature. The pellicles and glass fibre filters were placed in 6-well plates and 5 mL of 67% (v/v) sulfuric acid was added to each well on a rotatory shaker at room temperature to completely hydrolyze the cellulose. The total crystalline cellulose content in each pellicle fraction was estimated as glucose equivalents using an anthrone assay [30].

### DNA manipulations

DNA isolation from *K. xylinus* cultures was carried out using a modified protocol based on Murray and Thompson [37]. Briefly cells from a 50 mL bacterial culture were harvested by centrifugation and lysed with 500 μL of 2X cetyl trimethyl ammonium bromide (CTAB) buffer (2% (w/v), 1.4 M NaCl, 100 mM Tris-HCl pH 8.0, and 20 mM EDTA) containing 50 μg (w/v) RNaseA for 30 min at 65 °C. The lysed cells were cooled to room temperature then an equal volume of chloroform was added and the contents were mixed by inversion. The solution was centrifuged for 10 min at 17,000 x *g* to separate the aqueous and organic phases. The upper aqueous phase was transferred to a fresh tube and mixed with an equal volume of 2-propanol, then centrifuged for 20 min at 7000 x *g* to pellet the DNA. The pellet was washed with 70% ethanol by centrifugation at 7000 x *g* for 5 min. The ethanol layer was removed and the pellet was air dried for 15 min. Finally, the pellet was re-suspended in 100 μL of TE buffer (10 mM Tris HCl pH 8.0, 1 mM EDTA).

The *bcs* operon genes *bcsA*, *bcsB*, *bcsC*, and *bcsD* from wild type and *plr15* cultures were amplified using a touchdown PCR protocol using oligonucleotide primers designed according to the published cellulose synthase operon sequence of *K. xylinus* ATCC 53582 (GenBank accession number X54676.1). The parameters for touchdown PCR were: 98 °C for 2 min, followed by 10 cycles of 98 °C for 30s, 60 °C (decreasing by 1 °C/cycle) for 30s, and 72 °C for 3 min; followed by 30 cycles of 98 °C for 10s, 56 °C for 30s, and 72 °C for 3 min with a final extension at 72 °C for 5 min. Primer pairs used for amplification and DNA sequencing are listed in Additional file 3.

### Site-directed mutagenesis and allele replacement

The *bcsA* gene was PCR-amplified with high fidelity Phusion polymerase (New England Biolabs) using wild type genomic DNA as template. The PCR primers used for this amplification were BcsA *Bam*HI Frwd (5′- TACGGATCCAACGAAGAAGAATCCTAAGGC-3′) and BcsA *Spe*I Rev. (5′-ATCACTAGTGACGGGTTGTTCGTATCGT-3′). These primers introduced a 5′ *Bam*HI site and a 3′ *Spe*I site on the ends of the PCR product. PCR conditions for this amplification were: 98 °C for 2 min, followed by 10 cycles of 98 °C for 30 s, 60 °C (decreasing by 1 °C/cycle) for 45 s, 72 °C for 2 min and 30 s, followed by 30 cycles 98 °C for 10 s, 56 °C for 45 s, and 72 °C for 2 min and 30 s with a final extension at 72 °C for 5 min.

The *bcsA* PCR fragment was subsequently blunt-end cloned into the *Eco*RV site of pZErO-2 (Invitrogen). The resulting plasmid, named pZErO-2 *bcsA*, was sequenced to confirm the correct sequence of the *bcsA* gene was present. The *bcsA* fragment was then subcloned using the engineered *Bam*HI and *Spe*I sites into the suicide plasmid vector pKNG101, which was later used for allele replacement. The new plasmid, pKNG101 *bcsA* ColE1 *ori*, contained the ColE1 origin of replication from pZErO-2 in addition to the endogenous R6Kγ *ori* origins of replication; neither origin of replication allows for plasmid replication in *K. xylinus*. The plasmid sequence was confirmed by sequencing (Additional file 4).

Site directed mutagenesis of the *bcsA* gene was performed by substituting the GCC codon at position 1345 of the coding sequence corresponding to A449 with ACC, which codes for threonine. This was done using the forward primer BcsA Ala1345Thr Fwrd (5′ CATGTTCCACACCGTCGGCACG-3′) and reverse primer BcsA Ala1345Thr Rev. (5′-TGCGGGATGGCATAGGCC-3′). The PCR reaction conditions were: 98 °C for 2 min, followed by 25 cycles of 98 °C for 30s, 60 °C for 45 s, and 72 °C for 2.5 min and a final extension at 72 °C for 5 min. A 10X Kinase-Ligase-*Dpn*I Mix (10X KLD) was then used for phosphorylation, intramolecular circularization of the PCR product, and removal of the template as per manufacturer’s directions (New England Biolabs). The construct was transformed using high-efficiency DH5α competent cells. The presence of the mutation was confirmed by DNA sequencing.

The pKNG101 *bcsA* ColE1 *ori* variants were used for allele replacement of the endogenous *bcsA* gene. The *sacB* gene on the pKNG101 plasmid encodes a levansucrase enzyme which in several genera of gram-negative bacteria has been shown to be lethal in the presence of 5% sucrose [31]. This property facilitated selection for the integration of the mutated *bcsA* gene into the chromosome and for the excision of the vector. Transformation of *K. xylinus* with pKNG101 *bcsA* ColE1 *ori* resulted in the appearance of streptomycin-resistant transformants (Sm^R^) from the first homologous recombination. A total of 30 independent streptomycin-resistant transformants obtained from electroporation of *K. xylinus* wild type cells with the pKNG101 *bcsA* ColE1 *ori* construct were grown in SH medium with shaking (150 rpm) at 30 °C overnight. A volume of 150 μL of diluted cultures was plated on SH medium supplemented with 5% sucrose. A total of 400 sucrose-tolerant colonies were then grown in 200 μL SH medium containing 30 μM pellicin. A total of 8 pellicle-positive clones were obtained using this procedure. Genomic DNA from pellicle-positive and pellicle-negative colonies was extracted using the CTAB method described above. DNA sequencing of PCR products generated from both positive and negative clone genomic DNA was used to confirm the presence or absence of the site-directed mutation in the *bcsA* gene.

### Scanning electron microscopy

Pellicles were prepared for electron microscopy as previously described [29]. Observations were made using a Hitachi FlexSEM 1000 scanning electron microscope.

### In vitro cellulose synthase assays and ^14^[C]-glucose incorporation into cellulose

Cellulose synthase assays using crude membrane preparations were performed as previously described [29]. Radioactive ^14^[C]-glucose incorporation into cellulose of *K. xylinus* cultures were carried out by inoculating 1 mL of SH medium containing 1% glucose with cells to an OD_600_ = 0.5. Cultures were incubated for 2 h at 30 °C under static conditions, followed by addition of 5 μCi of ^14^[C]-glucose and incubated for an additional 5 h under the same conditions. Cells were harvested by centrifugation and washed 3 times with 1 mL fresh SH medium. To each pellet, 1 mL Updegraff solution [30] and incubated in a boiling water bath for 1 h. Insoluble material was collected onto Whatman GF/A glass fibre filters and the flow-through collected in fresh tubes. Filters were then washed 3 times with 4 mL water and once with 4 mL methanol. Filters were allowed to dry overnight at room temperature. The total activity of soluble and insoluble material was determined using a liquid scintillation counter.

### Statistical analysis

All statistics were performed using SigmaPlot 12.5 software. A student’s t-test or a one-way ANOVA test were performed for statistical analysis, and values were.

determined to be significant at a *P* value of < 0.05. A Saphiro-Wilk test was run in.

parallel to the one-way ANOVA test with *P* values for normality and equal variance set up at 0.050 in order to determine homogeneity of variance.

### Protein modelling

Structural analysis of *K. xylinus* BcsA was performed using the Phyre2 Protein Fold Recognition Server [38] based on the *Rhodobacter sphaeroides* cellulose synthase template PDB 4hg6A, to model a cellulose translocation intermediate of cellulose synthase subunit A. The pdb files created from these models were visualized using PyMOL software [39].

### Powder X-ray diffraction

Sample preparation for X-ray diffraction (XRD) to assess the crystallinity of cellulose was as previously described [29]. Briefly, samples were prepared by collecting pellicles of *K. xylinus* cultures grown in SH medium containing DMSO or by centrifugation (in the case of wild type) or pellicles (in the case of *plr* mutants) of cultures grown in the presence of 10 μM pellicin. Lyophilized material was finely ground, passed through a #60-gauge mesh and examined by Powder X-ray diffraction. X-ray diffractograms were recorded with a Philips PW3710 with Cu Ka radiation (1.54060 Å) with the generator working at 10 kV and 10 mA. Angular scanning was at a 2 θ step size of 0.020, a step time of 2.5 s. The relative crystallinity index (RCI) was calculated according to Segal et al. [40] using the formula RCI = *(I*_*t*_*-I*_*a*_*/I*_*t*_*) × 100*, where *I*_*t*_ is the total intensity of the (110) peak for cellulose Iα at 22.73° 2θ 22° and, for wild type culture with pellicin, of the (020) peak for cellulose II at 21.73° 2θ. The *I*_*a*_ is the amorphous intensity at 18° 2θ for cellulose Iα and 16° 2θ for cellulose II (Additional file 5 a). Peak identity was confirmed by comparison to diffraction patterns of cellulose Iα [41] using Match! Software (Crystal Impact, Germany).

## Additional files

Additional file 1: Effect of pellicin on growth of wild type and *plr15*. The viability of wild type (WT) and *plr15* cells was not affected by pellicin, but pellicin increases the final optical density of the wild type. Agitated cultures were grown at 30 °C in SH broth containing 0.3% (v/v) cellulase and either 30 μM pellicin (O) or DMSO (Δ) as a control. Values are means ± standard error for eight technical replicates. (TIF 67 kb)

Additional file 2: Representative pellicle formation in wild type and *plr* mutants. Pellicles formed after 4 days incubation at 30 °C in the presence of 10 μM pellicin (a), 50 μM pellicin (b) or 100 μM pellicin (c). The *bcsA*^*A449V*^ mutant shows more rapid pellicle confluence than the *plr15* or *bcsA*^*A449T*^ mutants. Pictures are representative of 3 replicates grown under the same conditions. (TIF 5475 kb)

Additional file 3: Primers used for *bcs* operon gene amplification and for sequencing of *bcs* genes. (XLSX 9 kb)

Additional file 4: Diagram showing the pKNG101_bcsA construct used for allele replacement in *K. xylinus*. (TIF 135 kb)

Additional file 5: Representative diffractograms of wild type and mutant pellicles of *K. xylinus*. In (a) positions of crystalline peaks matched to diffraction patterns of crystalline cellulose I α (green lines). In (b) overlay of diffractograms of pellicles from untreated and pellicin treated cultures of different *K. xylinus* genotypes. (TIF 135 kb)

## Data Availability

The datasets used and/or analysed during the current study available from the corresponding author on reasonable request.

## Abbreviations

BC: Bacterial cellulose
CBD: Carbohydrate binding domain
c-di-GMP: 3′,5′-cyclic diguanylate
CS: Cellulose synthase
CSC: Cellulose synthesizing complex
CTAB: Cetyl trimethyl ammonium bromide
DMSO: Dimethyl sulfoxide
EDTA: Ethylenediaminetetraacetic acid
EMS: Ethyl methane sulfonate
GT: Glycosyltransferase
KLD: Kinase-ligase-DpnI buffer
PCR: Polymerase chain reaction
TC: Terminal complex
TE: Tris-EDTA buffer
TMH: Transmembrane helix
XRD: X-ray diffraction

## References

[1] Brown RM (1990). Algae as tools in studying the biosynthesis of cellulose, natures most abundant macromolecule. Cell Walls and Surfaces, Reproduction, Photosynthesis.

[2] Brown RM, Willison JH, Richardson CL (1976). Cellulose biosynthesis in Acetobacter xylinum: visualization of the site of synthesis and direct measurement of the in vivo process. Proc Natl Acad Sci U S A.

[3] Delmer DP (1999). CELLULOSE BIOSYNTHESIS: Exciting times for a difficult field of study. Annu Rev Plant Physiol Plant Mol Biol.

[4] Festucci-Buselli RA, Otoni WC, Joshi CP (2007). Structure, organization, and functions of cellulose synthase complexes in higher plants. Brazilian J Plant Physiol.

[5] Park S, Baker JO, Himmel ME, Parilla PA, Johnson DK (2010). Cellulose crystallinity index: measurement techniques and their impact on interpreting cellulase performance. Biotechnol Biofuels.

[6] Nishiyama Y, Langan P, Chanzy H (2002). Crystal structure and hydrogen-bonding system in cellulose Iβ from synchrotron X-ray and neutron Fiber diffraction. J Am Chem Soc.

[7] Oehme DP, Doblin MS, Wagner J, Bacic A, Downton MT, Gidley MJ (2015). Gaining insight into cell wall cellulose macrofibril organisation by simulating microfibril adsorption. Cellulose..

[8] Saxena IM, Brown RM (2005). Cellulose biosynthesis: current views and evolving concepts. Ann Bot.

[9] Saxena IM, Kudlicka K, Okuda K, Brown RM (1994). Characterization of genes in the cellulose-synthesizing operon (acs operon) of Acetobacter xylinum: implications for cellulose crystallization. J Bacteriol.

[10] Morgan JLW, Strumillo J, Zimmer J (2013). Crystallographic snapshot of cellulose synthesis and membrane translocation. Nature..

[11] Saxena IM, Brown RM, Morohoshi N, Komamine A (2001). Biosynthesis of Cellulose.

[12] Zaar K (1979). Visualization of pores (export sites) correlated with cellulose production in the envelope of the gram-negative bacterium Acetobacter xylinum. J Cell Biol.

[13] Cannon RE, Anderson SM (1991). Biogenesis of bacterial cellulose. Crit Rev Microbiol.

[14] Mehta K, Pfeffer S, Brown RM (2014). Characterization of an acsD disruption mutant provides additional evidence for the hierarchical cell-directed self-assembly of cellulose in Gluconacetobacter xylinus. Cellulose..

[15] Omadjela O, Narahari A, Strumillo J, Melida H, Mazur O, Bulone V (2013). BcsA and BcsB form the catalytically active core of bacterial cellulose synthase sufficient for in vitro cellulose synthesis. Proc Natl Acad Sci U S A.

[16] Kawano S (2002). Cloning of cellulose synthesis related genes from Acetobacter xylinum ATCC23769 and ATCC53582: comparison of cellulose synthetic ability between strains. DNA Res.

[17] Nobles DR, Brown RM (2007). Many paths up the mountain: tracking the evolution of cellulose biosynthesis. Cellulose: molecular and structural biology.

[18] Amikam D, Galperin MY (2006). PilZ domain is part of the bacterial c-di-GMP binding protein. Bioinformatics..

[19] Morgan JLW, McNamara JT, Zimmer J (2014). Mechanism of activation of bacterial cellulose synthase by cyclic di-GMP. Nat Struct Mol Biol.

[20] Oglesby LL, Jain S, Ohman DE (2008). Membrane topology and roles of Pseudomonas aeruginosa Alg8 and Alg44 in alginate polymerization. Microbiology..

[21] Keiski C-L, Harwich M, Jain S, Neculai AM, Yip P, Robinson H (2010). AlgK is a TPR-containing protein and the periplasmic component of a novel exopolysaccharide secretin. Structure..

[22] Hu SQ, Gao YG, Tajima K, Sunagawa N, Zhou Y, Kawano S (2010). Structure of bacterial cellulose synthase subunit D octamer with four inner passageways. Proc Natl Acad Sci U S A.

[23] Iyer PR, Catchmark J, Brown NR, Tien M (2011). Biochemical localization of a protein involved in synthesis of Gluconacetobacter hansenii cellulose. Cellulose..

[24] Sajadi E, Babaipour V, Deldar AA, Yakhchali B, Fatemi SS-A (2017). Enhancement of crystallinity of cellulose produced by Escherichia coli through heterologous expression of bcsD gene from Gluconacetobacter xylinus. Biotechnol Lett.

[25] Kawano S, Tajima K, Kono H, Numata Y, Yamashita H, Satoh Y (2008). Regulation of endoglucanase gene (cmcax) expression in Acetobacter xylinum. J Biosci Bioeng.

[26] Nakai T, Nishiyama Y, Kuga S, Sugano Y, Shoda M (2002). ORF2 gene involved in the construction of high-order structure of bacterial cellulose. Biochem Biophys Res Commun.

[27] Deng Y, Nagachar N, Xiao C, Tien M, Kao T (2013). Identification and characterization of non-cellulose-producing mutants of Gluconacetobacter hansenii generated by Tn5 transposon mutagenesis. J Bacteriol.

[28] Tonouchi N, Tahara N, Kojima Y, Nakai T, Sakai F, Hayashi T (1997). A beta-glucosidase gene downstream of the cellulose synthase operon in cellulose-producing Acetobacter. Biosci Biotechnol Biochem.

[29] Strap JL, Latos A, Shim I, Bonetta DT (2011). Characterization of pellicle inhibition in Gluconacetobacter xylinus 53582 by a small molecule, Pellicin, identified by a chemical genetics screen. PLoS One.

[30] Updegraff DM (1969). Semimicro determination of cellulose inbiological materials. Anal Biochem.

[31] Kaniga K, Delor I, Cornelis GR (1991). A wide-host-range suicide vector for improving reverse genetics in gram-negative bacteria: inactivation of the blaA gene of Yersinia enterocolitica. Gene..

[32] Römling U, Galperin MY (2015). Bacterial cellulose biosynthesis: diversity of operons, subunits, products and functions. Trends Microbiol [Internet].

[33] McNamara JT, Morgan JLW, Zimmer J (2015). A molecular description of cellulose biosynthesis. Annu Rev Biochem.

[34] Benziman M, Haigler CH, Brown RM, White AR, Cooper KM (1980). Cellulose biogenesis: polymerization and crystallization are coupled processes in Acetobacter xylinum. Proc Natl Acad Sci U S A.

[35] Haigler CH, Brown RM, Benziman M (1980). Calcofluor white ST alters the in vivo assembly of cellulose microfibrils. Science..

[36] Harris DM, Corbin K, Wang T, Gutierrez R, Bertolo AL, Petti C (2012). Cellulose microfibril crystallinity is reduced by mutating C-terminal transmembrane region residues CESA1A903V and CESA3T942I of cellulose synthase. Proc Natl Acad Sci U S A.

[37] Murray MG, Thompson WF (1980). Rapid isolation of high molecular weight plant DNA. Nucleic Acids Res [Internet].

[38] Kelley LA, Mezulis S, Yates CM, Wass MN, Sternberg MJE (2015). The Phyre2 web portal for protein modelling, prediction and analysis. Nat Protoc [Internet].

[39] Schrodinger LLC. The PyMOL Molecular Graphics System, Version 1.8. 2015.

[40] Segal L, Creely JJ, Martin AE, Conrad CM (1959). An empirical method for estimating the degree of crystallinity of native cellulose using the X-ray diffractometer. Text Res J [Internet].

[41] French AD (2014). Idealized powder diffraction patterns for cellulose polymorphs. Cellulose [Internet].

