# Supplementary figures and images for: Missense mutations in a transmembrane domain of the Komagataeibacter xylinus BcsA lead to changes in cellulose synthesis
Source: BMC Microbiol. 2019 Sep 12;19:216. doi: 10.1186/s12866-019-1577-5 (PMC6740014; doi:10.1186/s12866-019-1577-5)

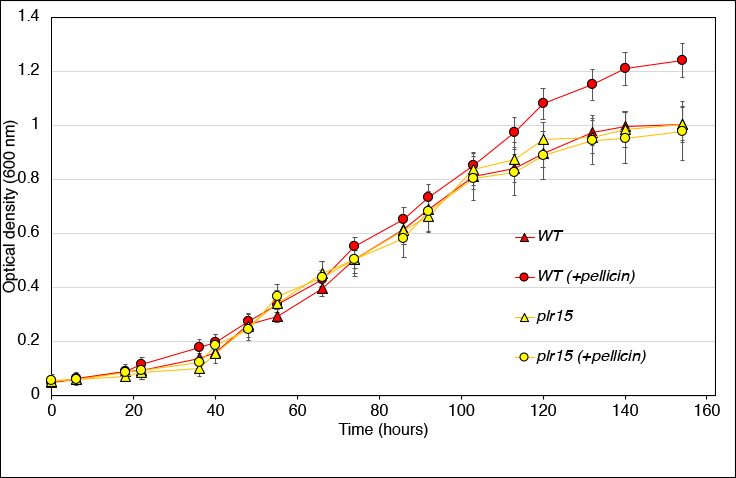

Supplement: Supplementary file 1 — Effect of pellicin on growth of wild type and plr15. The viability of wild type (WT) and plr15 cells was not affected by pellicin, but pellicin increases the final optical density of the wild type. Agitated cultures were grown at 30 °C in SH broth containing 0.3% (v/v) cellulase and either 30 μM pellicin (O) or DMSO (Δ) as a control. Values are means ± standard error for eight technical replicates. (TIF 67 kb) [file 12866_2019_1577_MOESM1_ESM.tif]

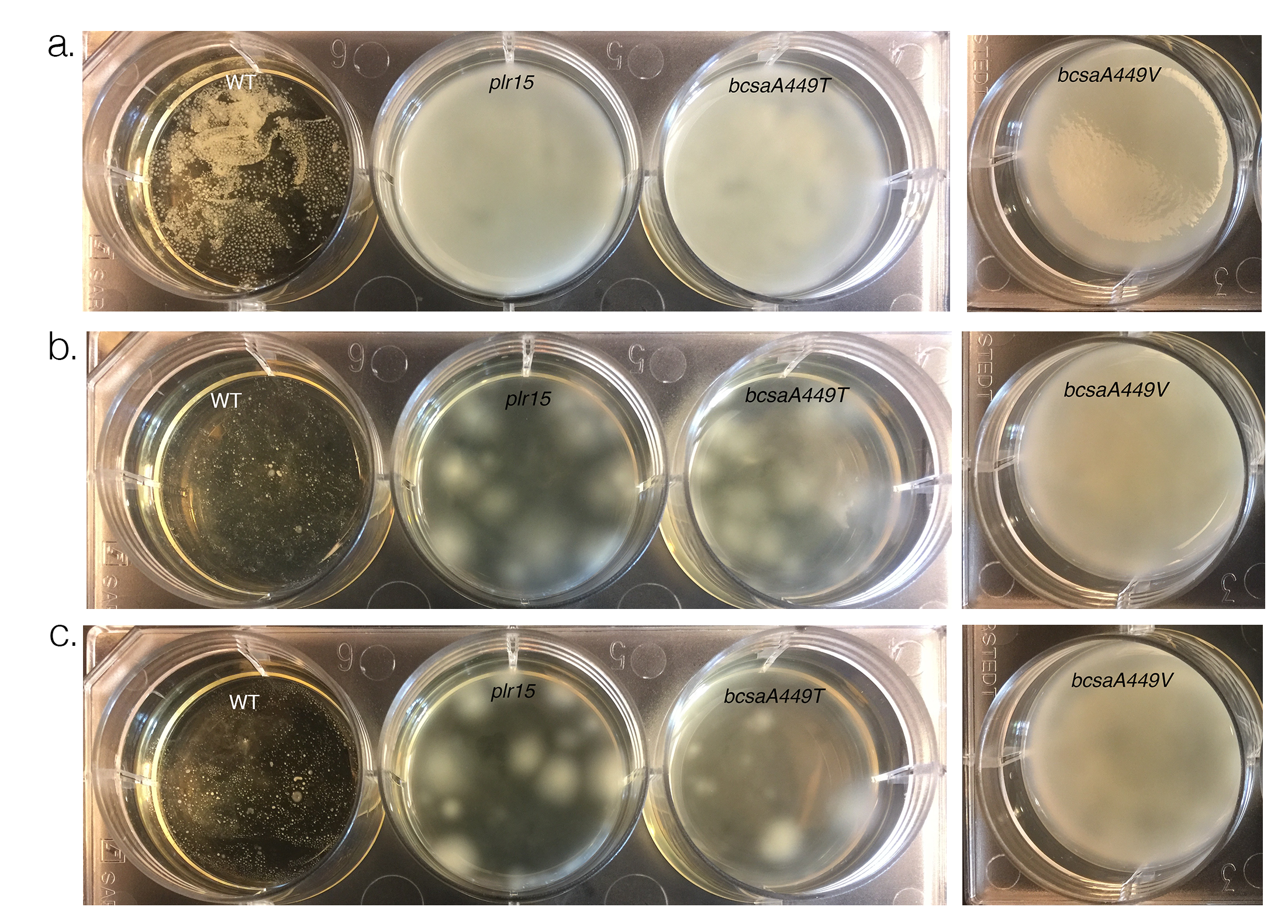

Supplement: Supplementary file 2 — Representative pellicle formation in wild type and plr mutants. Pellicles formed after 4 days incubation at 30 °C in the presence of 10 μM pellicin (a), 50 μM pellicin (b) or 100 μM pellicin (c). The bcsAA449V mutant shows more rapid pellicle confluence than the plr15 or bcsAA449T mutants. Pictures are representative of 3 replicates grown under the same conditions. (TIF 5475 kb) [file 12866_2019_1577_MOESM2_ESM.tif]

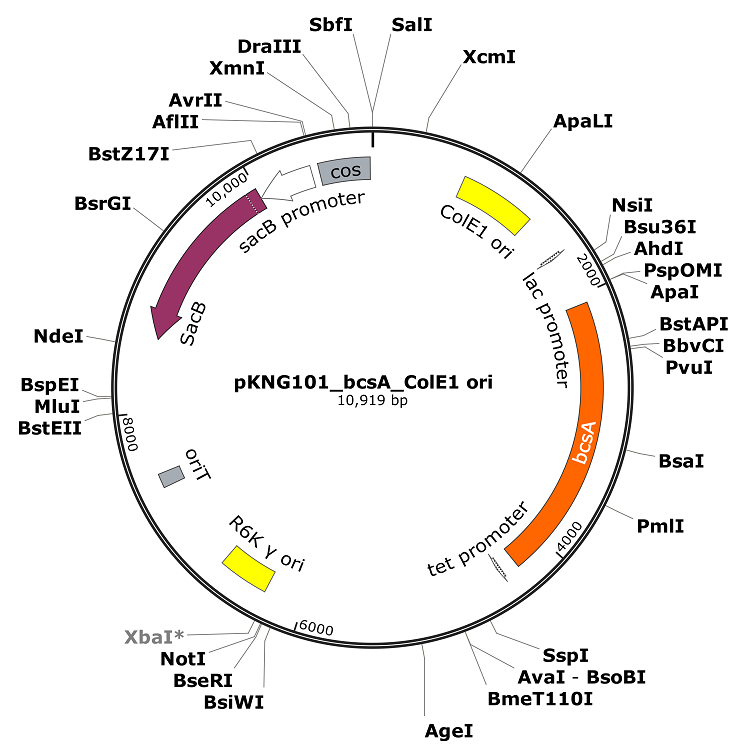

Supplement: Supplementary file 4 — Diagram showing the pKNG101_bcsA construct used for allele replacement in K. xylinus. (TIF 135 kb) [file 12866_2019_1577_MOESM4_ESM.tif]

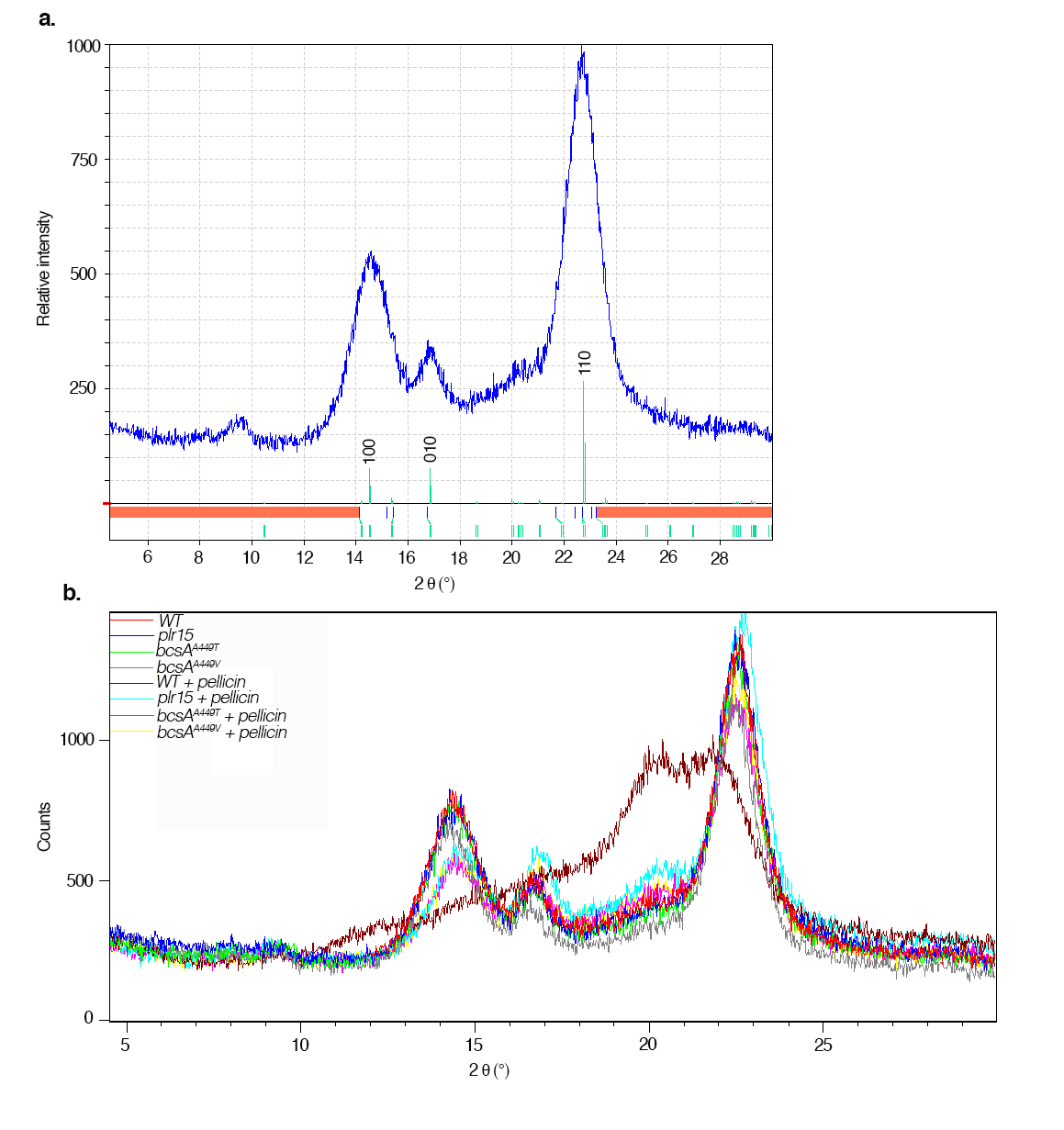

Supplement: Supplementary file 5 — Representative diffractograms of wild type and mutant pellicles of K. xylinus. In (a) positions of crystalline peaks matched to diffraction patterns of crystalline cellulose I α (green lines). In (b) overlay of diffractograms of pellicles from untreated and pellicin treated cultures of different K. xylinus genotypes. (TIF 135 kb) [file 12866_2019_1577_MOESM5_ESM.tif]
